# An Increase in Alpha Band Frequency in Resting State EEG after Electrical Stimulation of the Ear in Tinnitus Patients—A Pilot Study

**DOI:** 10.3389/fnins.2016.00453

**Published:** 2016-10-06

**Authors:** Marzena Mielczarek, Joanna Michalska, Katarzyna Polatyńska, Jurek Olszewski

**Affiliations:** ^1^Department of Otolaryngology, Laryngological Oncology, Audiology, and Phoniatrics, Medical University of LodzLodz, Poland; ^2^Department of Neurology, Polish Mother's Memorial Hospital Research InstituteLodz, Poland

**Keywords:** tinnitus, electrical stimulations, ear, neuromodulation, cortical activity

## Abstract

In our clinic invasive transtympanal promontory positive DC stimulations were first used, with a success rate of 42%. However, non-invasive hydrotransmissive negative DC stimulations are now favored, with improvement being obtained in 37.8% directly after the treatment, and 51.3% in a follow up 1 month after treatment. The further improvement after 1 month may be due to neuroplastic changes at central level as a result of altered peripheral input. The aim of the study was to determine how/whether a single electrical stimulation of the ear influences cortical activity, and whether changes observed in tinnitus after electrical stimulation are associated with any changes in cortical activity recorded in EEG. The study included 12 tinnitus patients (F–6, M-6) divided into two groups. Group I comprised six patients with unilateral tinnitus - unilateral, ipsilateral ES was performed. Group II comprised six patients with bilateral tinnitus—bilateral ES was performed. ES was performed using a custom-made apparatus. The active, silver probe—was immersed inside the external ear canal filled with saline. The passive electrode was placed on the forehead. The stimulating frequency was 250 Hz, the intensity ranged from 0.14 to 1.08 mA. The voltage was kept constant at 3 V. The duration of stimulation was 4 min. The EEG recording (Deymed QEST 32) was performed before and after ES. The patients assessed the intensity of tinnitus on the VAS 1-10.

**Results:** In both groups an improvement in VAS was observed—in group I—in five ears (83.3%), in group II—in seven ears (58.3%). In Group I, a significant increase in the upper and lower limit frequency of alpha band was observed in the central temporal and frontal regions following ES. These changes, however, were not correlated with improvement in tinnitus. No significant changes were observed in the beta and theta bands and in group II. Preliminary results of our research reveal a change in cortical activity after electrical stimulations of the ear. However, it remains unclear if it is primary or secondary to peripheral auditory excitation. No similar studies had been found in the literature.

## Introduction

Tinnitus is one of the vaguest audiological and otological symptoms. It is defined as a phantom sound perception, which does not reflect any corresponding physical activity. Tinnitus had been a disturbing disorder since antiquity. Although, only peripheral—cochlear pathology was initially considered an ignition site, it appeared that tinnitus was not always relieved after cochlear nerve section (Baguley et al., 2002). Later, Jastreboff's neurophysiological model of tinnitus proposed that the cochlea played a role in phantom sound generation, with the wide involvement of the central nervous system (Jastreboff, 1990). Recently, the development of such medical diagnostic methods as neurophysiological analysis and neuroimaging has stimulated the formulation of new hypotheses concerning tinnitus generation (Kaltenbach, 2011) and consequently, new original treatment modalities for patients.

In our clinic electrical stimulation (ES) of the ear in tinnitus treatment was first used in the 1980s. Invasive transtympanal promontory positive DC stimulations were first used, with a success rate of 42% (improvement in questionnaire by at least 4 points in 10 point scale) (Konopka et al., 2001). However, non-invasive hydrotransmissive DC stimulations are now favored, with improvement being obtained in 37.8% directly after the treatment, and 51.3% in a follow up 1 month after treatment (improvement in questionnaire by at least 20%) (Mielczarek and Olszewski, 2014). The improvement in tinnitus was accompanied by an improvement in hearing threshold in pure tone audiometry, indicating the condition of the peripheral hearing organ had improved. We hypothesized that further improvement after 1 month may be due to neuroplastic changes at central level as a result of altered peripheral input. In consequence of abovementioned hypothesis we posed a question about the possible mechanisms in which ear ES influences tinnitus: is it only peripheral (cochlear) activity modulation, or central—secondarily to peripheral alternation, or maybe purely central? Hypothetically, for electric current applied to the ear, soft tissues work as a conductor, thus none of the mechanisms can be excluded. There are many studies on the effect of ear ES on peripheral structures, however, to our knowledge, there is no study exploring alternations at a central cortical level directly after peripheral non-invasive ear ES.

Many studies have reported the suppression of tinnitus after cochlear implantation (CI) (Quaranta et al., 2004; Todt et al., 2015; Mertens et al., 2016), but the exact mechanism which is behind tinnitus suppression remains unclear as well. Zeng et al. noticed an increase in alpha power after low-rate electric stimulation via CI (Zeng et al., 2011). High-rate stimulation (5000 pps), however, appeared to be inefficient in inducing central activity (Middlebrooks, 2008) although restored activity at the auditory nerve was observed (Rubinstein et al., 2003).

In the literature, there are few explanations of how ear ES influences tinnitus (an increase in the neurotransmitter's transmission in the synapses—Latkowski, 1981, modification of the hearing organ's electrical potentials—Portmann et al., 1979, or improvement in the blood flow in the inner ear and synchronization of spontaneous impulses in the auditory nerve fibers—Watanabe et al., 1997) but none of them point to central mechanisms.

Thanks to animal models of peripheral tinnitus (induced by acoustic trauma or ototoxic drugs) the mechanisms of direct cochlear stimulation had been explained (Nuttall and Ren, 1995; Ren and Nuttall, 1995). Furthermore, animal models show correlations between a change in central activity as a result of peripheral cochlear modulations. Norena et al. noticed that ES of the cochlea in guinea pigs using a positive current decreased the spontaneous firing rate in the high characteristic frequency neurons of the inferior colliculi, while a negative current had the opposite effect. Such an effect was absent or reverse for low characteristic frequency neurons (Noreña et al., 2015). Mulders et al. obtained reduction of pathological hyperactivity in inferior colliculi after intraperitoneal or cochlear injection of furosemide (Mulders et al., 2014a). What's more, the authors observed similar results after chronic furosemide treatment without hearing impairment (Mulders et al., 2014b). Reducing central hyperactivity authors claim to reduce behavioral signs of tinnitus decreasing spontaneous firing of auditory afferent.

Thanks to electrophysiological studies in tinnitus changes in cortical activity were widely explored (Shulman and Goldstein, 2002; Weisz et al., 2005; Adjamian et al., 2012; Meyer et al., 2014; Schlee et al., 2014). In consequence treatment methods aiming at neuromodulation are now “mainstream therapies.” Thus neurophysiological measurements like EEG, MEG serve as an objective outcome measure to quantify therapeutic benefit.

The aim of the study was to determine how/whether a single electrical stimulation of the ear influences cortical activity, and whether changes observed in tinnitus after electrical stimulation are associated with any changes in cortical activity recorded in EEG.

## Materials and methods

The study included 12 tinnitus patients (F–6, M-6) divided into two groups. Group I comprised six patients with unilateral tinnitus (six tinnitus ears, five—left, one—right ear), aged 36–73 (average 58.50 years, SD 11.83 years), unilateral, ipsilateral ES was performed. Group II comprised six patients with bilateral tinnitus (12 tinnitus ears), aged 38-78 years (average 53.50 years, SD 18.16 years), bilateral ES was performed starting with the right ear. The groups were balanced in terms of the age, and tinnitus duration.

Before the beginning of the study, ENT examination was performed, together with any necessary audiological and radiological diagnostics. The exclusion criteria included the following: pathology in the external and/or the middle ear, central nervous system disorders (e.g., epilepsy), positive history of neoplasms, implanted pacemaker. Patients with tinnitus in the head, not the ears, were disqualified from the research.

ES was performed using a custom-made apparatus supplied with four 1.5 V batteries. The device has an on / off button, frequency and current intensity buttons. The patient was in a lying position, on the side with the stimulated ear upwards. The external ear canal was filled with saline solution. The active, silver probe—was immersed inside the external ear canal, avoiding contact with the skin of the canal. The passive electrode was placed on the forehead in the midline (glabella) after skin abrasion with a suitable sterile abrasive electrode paste and clean gauze. The two electrodes were placed in such a way that the current could flow throughout the hypothetical plane (longitudinal axis) of the cochlea. Direct rectangular negative current was applied via the active electrode. In all cases, the stimulating frequency was 250 Hz, the intensity ranged from 0.14 to 1.08 mA, and was applied according to the sensation experience by the patient. The voltage was kept constant at 3 V. The duration of each stimulation was 4 min.

EEG (Deymed QEST 32) was conducted in a room that conforms to EEG standards. Two EEG recordings of 5 min were performed before and after ES (in group I—before and after unilateral ES, in group II—before and after bilateral ES). A Deymed TruScan (10/20 system) electro-cap was fitted. The impedance of the electrodes didn't depend on the head position. The patients were asked to close their eyes and relax. They were asked to refrain from drugs, such as caffeine or alcohol, for 24 h preceding the EEG recording. None of the patients were using drugs influencing CNS activity (e.g., benzodiazepines). The upper and lower limit of a frequency band (Hz) and an averaged wave amplitude (μV) were assessed for alpha, beta and theta oscillations. These parameters were assessed before and after electrical stimulation, furthermore these results were assessed with regard to tinnitus change (assessed in VAS).

Comparisons of the limit frequencies of the bands and averaged amplitudes for alpha, beta and theta were made between reading taken before and after ES. In every recording three samples of 1–3 s were chosen and next marked. The minimal and maximal frequencies and amplitudes were automatically counted by the system. In order to assess potential changes in cortical activity, we compared between the following regions: central frontal (right F4-C4, left F3-C3), temporo –occipital (right T6-O2, left T5-O1), parieto—occipital (right P4-O2, left P3-O1), temporal anterior (right F8-T6, left F7-T5), central (right C4-T4, left T3-C3), posterior (right T4-T6, left T3-T5) and the left and right hemisphere.

In addition, the patients were asked to assess the intensity of tinnitus on the visual analog scale (VAS 1–10) directly before and after electrical stimulation.

The study was approved by the Institutional Review Board of the Medical University of Lodz. All subjects gave written informed consent in accordance with the Declaration of Helsinki.

### Statistical analysis

The exact Mann-Whitney rank-sum test for independent data and the exact Wilcoxon matched-pairs signed-ranks test were used to fit data. The exact procedures were chosen due to the small sizes of the samples. Generalized estimating equations (GEE) with robust standard errors also were used when dealing with more than one independent variable, and when inclusive of repeated measures. A level of *p* < 0.05 was considered statistically significant.

## Results

Following ES, in Group I, an improvement in VAS was observed in five ears (83.3%)—tinnitus disappeared in two of them (33.3%) and no change was observed in the remaining ear (16.7%)—i.e., no deterioration in tinnitus was observed. In Group II, improvement was observed in seven ears (58.3%) and tinnitus disappeared in two of them (16.7%), while no change was observed in the other five ears (41.7%).

**Figure 1:**
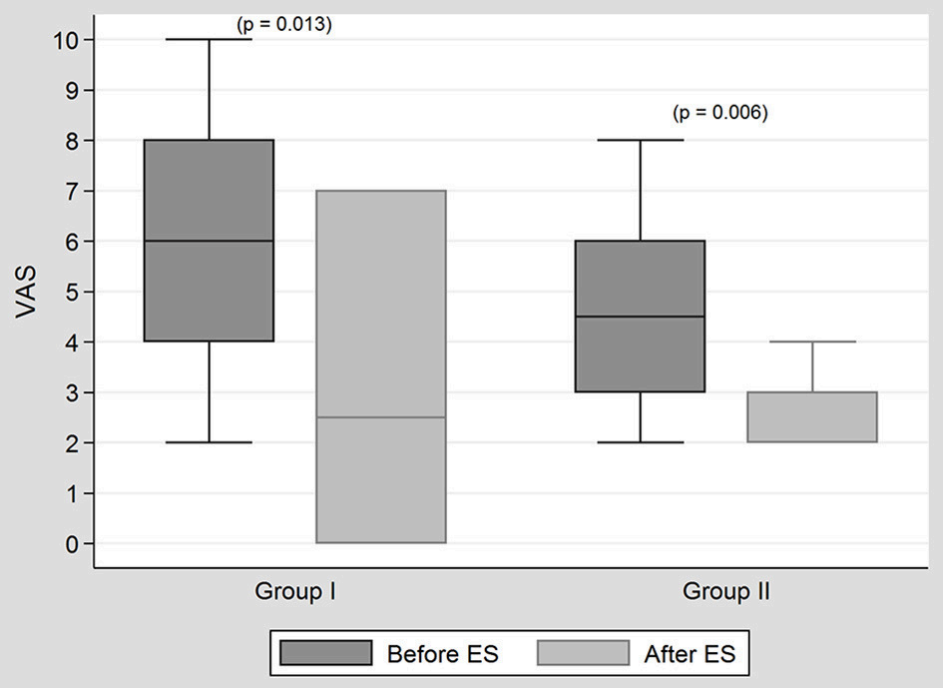
**The results of tinnitus treatment with ES in both groups**. In both groups, significant changes in tinnitus, given according to VAS, were noted after ES. However, no significant difference was observed between the two groups in terms of tinnitus improvement according to VAS (*p* = 0.699).

In both groups, significant changes in tinnitus, given according to VAS, were noted. However, no significant difference was observed between the two groups in terms of tinnitus improvement according to VAS (*p* = 0.699) (Figure 1).

**Figure 2:**
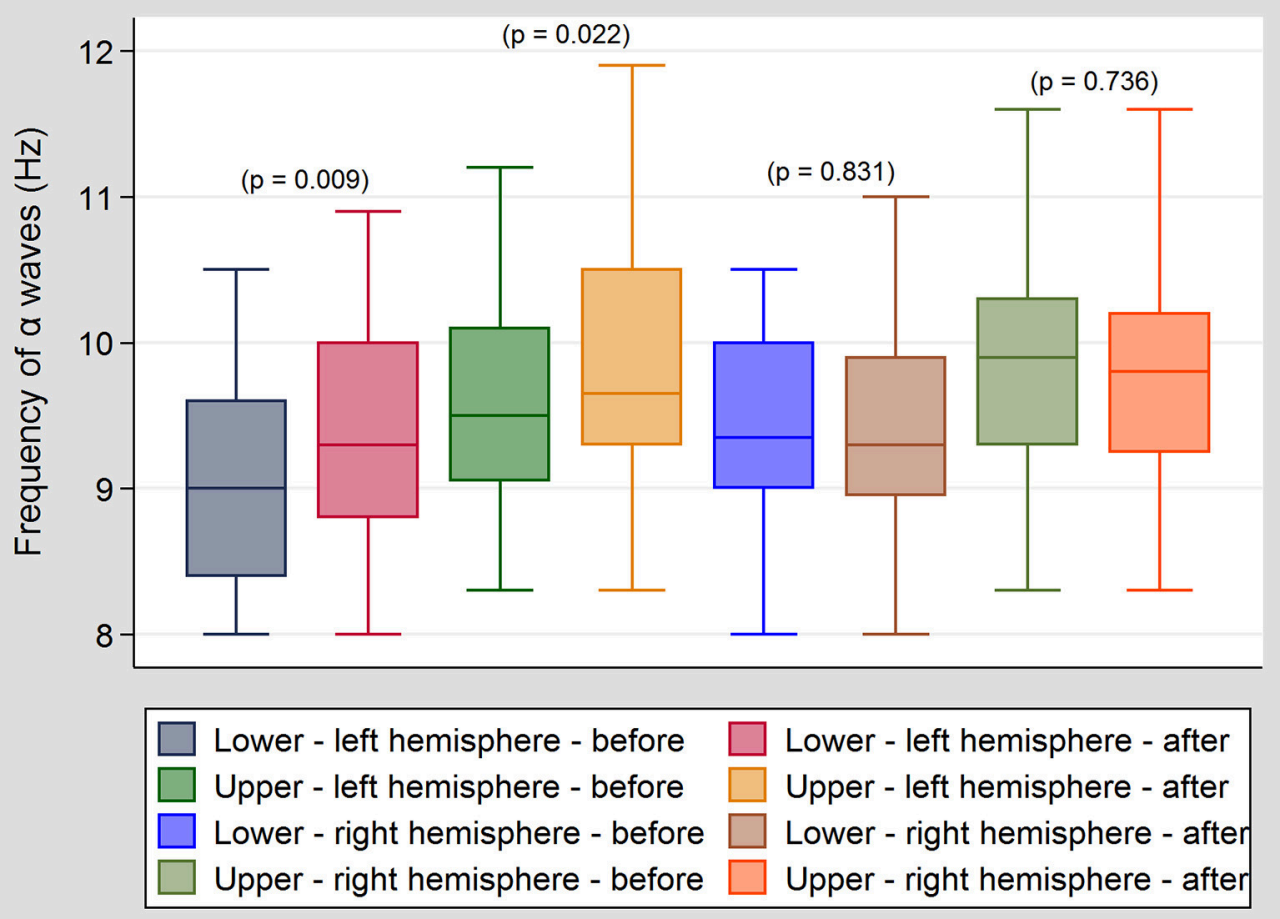
**Differences between upper and lower frequency of alpha band in group I before and after ES**. In Group I, a significant increase in the upper and lower limit of frequency of the alpha band was observed in the left central temporal and frontal regions following ES. No significant changes were observed in the beta and theta bands and the right side in this group.

In Group I, a significant increase in the upper and lower limit of frequency of the alpha band was observed in the left central temporal and left frontal regions following ES (Figure 2). These changes, however, were not correlated with any improvement in tinnitus. No significant changes were observed in the beta and theta bands and on the right side (hemisphere). In Group II, no significant changes were observed in the alpha, beta and theta bands. No significant changes were observed in the waves' amplitude (μV) before and after ES, and when right and left sides were compared. The intensity of current (mA) used in ES did not differ significantly (*p* = 0.772) between the groups (in both groups mean intensity was 0,47 mA). No pathological activity was observed in EEG after ES. We didn't observe gamma, nor delta oscillations in our patients.

## Discussion

Alpha oscillations are the dominant rhythm at rest in the sensory regions. This activity reflects excitatory—inhibitory balance, with decreased levels of alpha in excitatory conditions and increased in inhibition (Klimesch et al., 2007). Active stimulus processing or stimulus anticipation results in alpha power decrease which relates to decreased functional inhibition (Weisz et al., 2011). In consequence hyperexcitability within central auditory structures appears resulting from downregulation due to reduced peripheral—cochlear—activity. Increased spontaneous firing rate in neurons of central auditory pathway is hypothesized to be a crucial factor for tinnitus appearance (Noreña and Eggermont, 2003). Given the fact that changes in alpha band reflect shifts between excitatory—inhibitory processes and the view that tinnitus is a result of excitatory—inhibitory imbalance, in some research alpha rhythm and other frequency bands were assessed before and after tinnitus treatment. There are works on a change in alpha frequency, but most of the papers report changes in alpha power (reflecting neuronal synchronization).

In practice, two approaches in stimulations in tinnitus are possible (central vs. peripheral) and apparently both can directly or indirectly modify central activity. One approach - peripheral stimulation, aims to increase peripheral input, which results in spontaneous firing rate decrease and in consequence, in reducing central hyperactivity. The other - primarily aims to modify pathological central hyperactivity. Since tinnitus is known to be associated with excessive spontaneous activity in central auditory system (Mühlnickel et al., 1998; De Ridder et al., 2014), a spectrum of superficial brain stimulation techniques have been proposed to interfere with this maladaptive activity. EEG and MEG therefore would appear to be appropriate tools to quantify this pathological activity before and after implementation of therapeutic method.

Adamchic et al. Schlee et al. described reduced alpha frequency in tinnitus patients (Adamchic et al., 2014; Schlee et al., 2014). In the light of these results, our findings appear to be of advantage in tinnitus treatment. Some other studies, however, didn't repeat these findings (Shulman and Goldstein, 2002; Adjamian et al., 2012; Meyer et al., 2014). In terms of other oscillation bands the results are inconsistent as well. Schlee et al. demonstrated that shorter duration of tinnitus (less than 3 years) was correlated with a larger alpha variability than longer tinnitus duration (more than 3 years). The authors hypothesized that the latter may reflect decreased neuroplastic capacity of brain. Furthermore it was shown that alpha activity was variable and fluctuated with time in healthy subjects, while in tinnitus individuals this ability was significantly reduced. Interestingly, the authors interpreted alpha variability as an indicator for spontaneous tinnitus remission (Schlee et al., 2014). In our research, the changes in alpha frequency may hypothetically be indicative for better neuroplastic potential and thus better future therapeutic outcome. On the other hand, in the light of abovementioned research, the changes in alpha frequency might result just from physiological fluctuations (why they were absent in a group of bilateral tinnitus—remains for the authors unclear).

In our research significant changes in alpha band were observed in the frontal and temporal regions, both on the left side. This is probably due to ipsilateral ES (in most of the cases tinnitus was left sided). It is possible that a better balance between left and right sided tinnitus in the group would balance lateralization of the results. Weisz et al. (2005) observed marked changes in alpha and delta power in temporal (right) and frontal (left) regions and suggested that these regions might be involved in tinnitus-related cortical activity. Temporal region would then be associated with perceptual aspects of the sound, and frontal—with distress and attention of tinnitus. In this research, like in our research, the most of the patients had left sided tinnitus. The authors concluded that the lateralization of the results might vanish if the study groups were balanced in terms of left and right-sided tinnitus. The justification for this assumption was Jastreboff's neurophysiological model of tinnitus which considered prefrontal cortex a place where sensory and emotional aspects of tinnitus are integrated, but without reference to the lateralization (Jastreboff, 1990).

Since the pathological cortical activity (increase in γ / δ and decrease in alpha band activity) appears to reflect the fact of tinnitus generation and relating distress, some studies demonstrated normalization of these activities in a course of tinnitus treatment (Müller et al., 2013; Adamchic et al., 2014).

Thus three promising neuromodulation methods emerged: transcranial direct (tDCS) and alternating current simulation (tACS), and transcranial magnetic stimulation (TMS) (Zaehle et al., 2010; Song et al., 2012; Vanneste et al., 2013a). Depending on the polarity, tDCS increases or decreases cortical excitability (Miranda et al., 2006). Cathodal tDCS induces hyperpolarization of the cortex, while anodal tDCS intensifies excitation through depolarization of the neurons (Monte-Silva et al., 2013; Joos et al., 2014). tASC increases alpha power (Zaehle et al., 2010; Vossen et al., 2015) and intracotrical inhibition. Random noise stimulation (tRNS) (Van Doren et al., 2014; Joos et al., 2015)—a new transcranial stimulation technique has recently emerged and appeared to be more effective in tinnitus reduction when compared to tDCS and tACS (Vanneste et al., 2013b; Claes et al., 2014).

In the light of inconsistent and non-replicable data on spontaneous EEG activity in tinnitus patients, two papers (Adjamian and Pierzycki et al.) have recently appeared shedding new light on EEG / MEG measurements in tinnitus (Adjamian, 2014; Pierzycki et al., 2016). Adjamian points to a careful analysis and interpretation of EEG / MEG data in tinnitus patients. Neglecting factors unrelated to tinnitus (e.g., the use of non-standardized tools, or protocols) and comorbidities such as hearing loss, hyperacusis, stress or depression might be sources for the flaws in EEG/MEG research results (Adjamian, 2014). In other paper, the author demonstrated that there are no differences due to tinnitus in any frequency band, except possibly delta (however it remains unclear if it was the effect of hearing loss or tinnitus together with hearing loss; Adjamian et al., 2012).

Vanneste et al. demonstrated correlation between psychoacoustic aspect of tinnitus and cortical activity. An increase in tinnitus loudness was associated with an increase in gamma-band activity in the auditory cortex (Vanneste et al., 2013c), which was attributed by the authors to a thalamo-cortical dysrhythmia model (De Ridder et al., 2015). This states that in the deafferented state, the dominant resting state alpha rhythm decreases to theta band activity (Llinás et al., 1999) and that constant abnormal coupled theta/gamma band activity occurs as a consequence of hyperpolarization in thalamus nuclei (De Ridder et al., 2011). In contrast, Pierzycki et al. found no relationship between whole scalp EEG band powers and psychoacoustic or psychosocial variables of tinnitus. The authors concluded that resting state whole scalp EEG should not be used as a biomarker for tinnitus (Pierzycki et al., 2016).

Preliminary results of our research revealed an increase in alpha frequency in the left central temporal and frontal regions. Although, this effect might suggest the treatment has potential, the results indicate that it was not correlated with improvement in tinnitus. Since the literature data on EEG / MEG activity in tinnitus has been inconsistent so far, the relevance of the present findings is not clear and needs further research. In previous research we obtained tinnitus relief after treatment which involved 15 ES. It is possible that the key factor contributing to improvement was repeated stimulation which was needed to evoke and consolidate changes in central auditory system. At this stage, it is unclear whether modulation of cortical activity is primary or secondary to peripheral auditory excitation. At the time of writing, no similar studies had been found in the literature.

In the light of research on peripheral electrical stimulation and central stimulation techniques (current or magnetic stimulation) it appears that both approaches may be efficient in tinnitus treatment.

The limitations of the study are the small size of the groups and the lack of a placebo and control group. In addition, the reason why EEG changes were obtained only in the unilateral tinnitus patients (group I), despite improvement in tinnitus having been observed in both groups, needs further investigation. The exact correlations between changes in cortical activity and tinnitus reduction will be the subject of future research.

## Conclusions

Our results indicate that peripheral external ear ES changes cortical activity in tinnitus patients. One of the possible mechanisms in which ES influences tinnitus may therefore be a change in the cortical activity present within the left central temporal and frontal regions. However, whether this effect is primary or secondary to auditory system excitation remains to be investigated. Of the various forms of ES presented above, each may play a role in the tinnitus improvement observed in our study.

## Author contributions

MM, JM, KP, and JO: Substantial contributions to the conception, design of the work, the acquisition, analysis, or interpretation of data for the work, drafting the work, revising it critically for important intellectual content, final approval of the version to be published, agreement to be accountable for all aspects of the work in ensuring that questions related to the accuracy or integrity of any part of the work are appropriately investigated and resolved.

### Conflict of interest statement

The authors declare that the research was conducted in the absence of any commercial or financial relationships that could be construed as a potential conflict of interest.
